# Special Issue “Molecular Research in Retinal Degeneration”

**DOI:** 10.3390/ijms27031287

**Published:** 2026-01-28

**Authors:** Sandra Tenreiro, Francisco J. Diaz-Corrales

**Affiliations:** 1 iNOVA4Health, NOVA Medical School, Universidade NOVA de Lisboa, Campo Martires da Pátria 130, 1169-056 Lisboa, Portugal; 2 Department of Integrative Pathophysiology and Therapies, Andalusian Molecular Biology and Regenerative Medicine Centre (CABIMER), Junta de Andalucia, CSIC, Universidad de Sevilla, Universidad Pablo de Olavide, Avda. Americo Vespucio 24, 41092 Seville, Spain

Retinal diseases comprise a highly heterogeneous group of disorders that collectively represent a major cause of visual impairment and blindness worldwide [1,2,3]. Despite remarkable advances in clinical imaging and diagnostics, effective treatments for many retinal disorders remain limited, largely due to the extraordinary molecular, genetic, and cellular complexity of the retina [4,5,6]. A deeper molecular understanding of retinal homeostasis and degeneration is therefore essential to enable meaningful therapeutic translation.

This Special Issue of the International Journal of Molecular Sciences, entitled “Molecular Research in Retinal Degeneration”, brings together a collection of original research articles and reviews that exemplify current efforts to dissect retinal diseases at the molecular level while simultaneously advancing translational strategies (Table 1). The contributions highlight how genetic discoveries, mechanistic insights, and innovative human-relevant models are converging to address long-standing challenges in retinal research.

A central theme emerging from this Special Issue is the critical role of genetic heterogeneity and genotype–phenotype correlations in inherited retinal diseases. Comprehensive analyses of disease-causing variants in genes such as *ABCA4*, *USH2A*, and *POMGNT1* underscore not only the extensive allelic diversity underlying retinal degeneration but also the remarkable clinical variability associated with specific molecular defects in line with the conclusions of Dayma et al., Ogorodova et al., and Ziccardi et al. These studies emphasize that understanding disease severity, age of onset, and tissue specificity requires integrating molecular genetics with functional and clinical data, an approach that is increasingly relevant for patient stratification and precision medicine.

Beyond genetics, several contributions explore molecular and cellular mechanisms that govern retinal integrity and degeneration. Insights into complement signaling and endothelial barrier regulation highlight tissue-specific responses within the neurovascular system, reinforcing the concept that the retina possesses unique protective and regulatory properties as described by Wolf and Guempelein et al. Likewise, investigations into post-translational modifications, such as O-mannosylation, reveal how subtle disruptions in molecular pathways can lead to highly variable retinal phenotypes according to the findings reported by Ziccardi and colleagues. Together, these studies illustrate how molecular dysregulation intersects with cellular architecture to drive disease progression.

Equally important is the strong emphasis on advanced human-relevant experimental models. The application of human induced pluripotent stem cell (hiPSC) technology, combined with precise genome editing approaches such as prime editing, enables the generation of isogenic disease models that faithfully recapitulate patient-specific mutations as demonstrated by Cerna-Chavez and co-workers. Retinal organoids derived from hiPSCs further provide unprecedented opportunities to study human retinal development, degeneration, and therapeutic response in a three-dimensional context as highlighted in the work of Galindo and Caballano et al. These platforms not only deepen mechanistic understanding but also serve as powerful testbeds for evaluating gene- and cell-based interventions.

From a translational perspective, this Special Issue highlights emerging regenerative strategies aimed at restoring retinal function. Proof-of-concept studies demonstrating the survival, integration, and differentiation of transplanted retinal ganglion cell precursors underscore the therapeutic potential of cell replacement approaches for optic neuropathies and advanced retinal degeneration as demonstrated by Lei and Zhang et al. While significant hurdles remain, including immune modulation, functional integration, and long-term safety, such work represents an important step toward future restorative therapies.

Collectively, the contributions in this Special Issue underscore a key message: progress in retinal medicine increasingly depends on the integration of molecular biology, genetics, advanced modeling, bioengineering, and clinical expertise (Figure 1). This Special Issue exemplifies the type of integrative, molecularly driven, and multidisciplinary research efforts that are now being fostered by coordinated initiatives such as COST Action Retina4Future [7]. By promoting collaboration across disciplines and borders, such initiatives aim to accelerate the translation of molecular discoveries into early diagnosis, reliable biomarkers, and effective therapies for retinal diseases.

In conclusion, the studies presented here reflect both the complexity of retinal disorders and the innovative strategies being developed to address them. Continued interdisciplinary collaboration, coupled with molecular precision and translational focus, will be essential to transform fundamental discoveries into tangible clinical benefit. We hope that this Special Issue will stimulate further research, foster new collaborations, and contribute to shaping the future of retinal medicine.

## Figures and Tables

**Figure 1:**
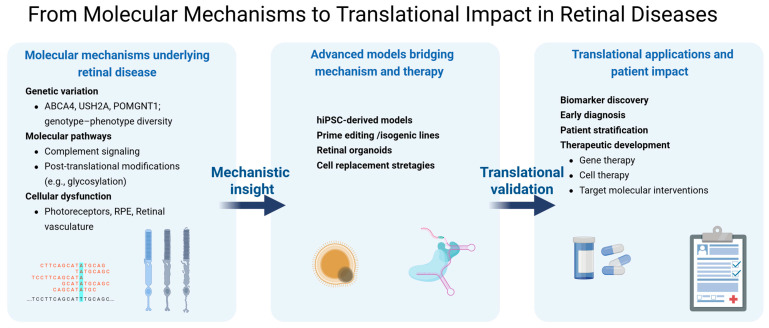
Integrative framework linking molecular mechanisms to translational outcomes in retinal diseases. Genetic heterogeneity and molecular pathway dysregulation inform the development of advanced human-relevant models, including hiPSC-based systems, genome editing approaches, retinal organoids, and regenerative strategies. These platforms enable biomarker discovery, patient stratification, and therapeutic innovation through multidisciplinary collaboration.

**Table 1:** List of the articles present in this Special Issue.

First Author(s)	Reference
Wolf, H.N. and Guempelein, L.	Wolf, H.N.; Guempelein, L.; Schikora, J.; Pauly, D. C3a Mediates Endothelial Barrier Disruption in Brain-Derived, but Not Retinal, Human Endothelial Cells. *Int. J. Mol. Sci.* **2024**, *25*. https://doi.org/10.3390/ijms252011240.
Ogorodova, N.	Ogorodova, N.; Stepanova, A.; Kadyshev, V.; Kuznetsova, S.; Ismagilova, O.; Chukhrova, A.; Polyakov, A.; Kutsev, S.; Shchagina, O. A Comparative Evaluation of the Genetic Variant Spectrum in the USH2A Gene in Russian Patients with Isolated and Syndromic Forms of Retinitis Pigmentosa. *Int. J. Mol. Sci.* **2024**, *25*. https://doi.org/10.3390/ijms252212169.
Lei, Q. and Zhang, R.	Lei, Q.; Zhang, R.; Yuan, F.; Xiang, M. Integration and Differentiation of Transplanted Human iPSC-Derived Retinal Ganglion Cell Precursors in Murine Retinas. *Int. J. Mol. Sci*. **2024**, *25*. https://doi.org/10.3390/ijms252312947.
Cerna-Chavez, R.	Cerna-Chavez, R.; Ortega-Gasco, A.; Baig, H.M.A.; Ehrenreich, N.; Metais, T.; Scandura, M.J.; Bujakowska, K.; Pierce, E.A.; Garita-Hernandez, M. Optimized Prime Editing of Human Induced Pluripotent Stem Cells to Efficiently Generate Isogenic Models of Mendelian Diseases. *Int. J. Mol. Sci.* **2025**, *26*. https://doi.org/10.3390/ijms26010114.
Galindo-Cabello, N. and Caballano- Infantes, E.	Galindo-Cabello, N.; Caballano-Infantes, E.; Benites, G.; Pastor-Idoate, S.; Diaz-Corrales, F.J.; Usategui-Martín, R. Retinal Organoids: Innovative Tools for Understanding Retinal Degeneration. *Int. J. Mol. Sci.* **2025**, *26*, 1–24. https://doi.org/10.3390/ijms26073263.
Ziccardi, L.	Ziccardi, L.; Barbano, L.; D’Andrea, M.; Bruselles, A.; Dell’Aquila, C.; Niceta, M.; Mancini, C.; Leone, A.; Carvetta, M.; Albanese, M.; et al. Variable Ophthalmologic Phenotypes Associated with Biallelic Loss-of-Function Variants in POMGNT1. *Int. J. Mol. Sci.* **2025**, *26*. https://doi.org/10.3390/ijms26073278.
Dayma, K.	Dayma, K.; Rajanala, K.; Upadhyay, A. Stargardt’s Disease: Molecular Pathogenesis and Current Therapeutic Landscape. *Int. J. Mol. Sci*. **2025**, *26*, 1–23. https://doi.org/10.3390/ijms26147006.
